# Symptomatic breast services in Ireland: how do they compare with national and international standards?

**DOI:** 10.1186/bcr2948

**Published:** 2011-11-04

**Authors:** D O'Leary, L Rainford

**Affiliations:** 1 UCD, School of Medicine and Medical Science, Dublin, Ireland

## Introduction

A study of symptomatic breast units geographically spread over Ireland collected image quality, compression and radiation dose data from 18 mammography units; so how do these optimisation parameters compare nationally and internationally? The mean glandular dose (MGD) diagnostic reference level was proposed for the all-digital breast screening service [1] but not for the symptomatic breast service.

## Methods

The quantitative and qualitative data were analysed using SPSS. Recommendations of MGD diagnostic reference levels were made at various levels for film-screen mammography (FSM) and full-field digital mammography (FFDM) units to match those levels published in worldwide.

## Results

MGDs received by symptomatic breast patients within Ireland are higher than those received in the all-digital Irish Breast Screening service, although the differences for FFDM are not substantial; 55 to 65 mm breast: 1.75 mGy (screening) versus 2.4 mGy (symptomatic) at the 95th percentile. The four-view routine mammography MGDs obtained in symptomatic breast units in Ireland are, however, substantially different from other screening units with mixed FSM/FFDM modalities: 4.5 mGy (UK); 4.98 mGy (USA) versus 5.96 mGy (FFDM, symptomatic) and 9.63 mGy (FSM, symptomatic). Various reasons are proposed for the differences.

## Conclusion

MGD diagnostic reference levels achieved in the screening service may be lower due to the exacting requirements for radiographer training, nonsurgical alteration of patient breasts and equipment quality assurance levels. Greater training of radiographers performing mammography in the symptomatic breast services is required to standardise mammographic projections with regard to MGDs delivered.
